# Positive Impact on Physical Activity and Health Behaviour Changes of a 15-Week Family Focused Intervention Program: “Juniors for Seniors”

**DOI:** 10.1155/2016/5489348

**Published:** 2016-09-27

**Authors:** Michał Bronikowski, Małgorzata Bronikowska, Beata Pluta, Janusz Maciaszek, Maciej Tomczak, Agata Glapa

**Affiliations:** ^1^Department of Didactics of Physical Activity, University School of Physical Education, Krolowej Jadwigi 27/38, 61-871 Poznan, Poland; ^2^Department of Recreation, University School of Physical Education, Krolowej Jadwigi 27/38, 61-871 Poznan, Poland; ^3^Department of Physical Activity Study and Health Promotion, University School of Physical Education, Krolowej Jadwigi 27/38, 61-871 Poznan, Poland; ^4^Department of Psychology, University School of Physical Education, Krolowej Jadwigi 27/38, 61-871 Poznan, Poland; ^5^Physical Activity, Sport and Recreation Focus Area, North-West University, Private Bag Box X6001, Potchefstroom 2520, South Africa

## Abstract

The promotion of physical activity (PA) in children and their parents requires effective planning and sometimes even interventions. This study shows the effect of PA during a 15-week intervention program “Junior for Seniors” by applying a socioecological model to the interpretation of the data. This comprehensive approach emphasizes the fact that health promotion should focus not only on intrapersonal factors but also on the multilevel factors that might be determinants and modulators of increased PA. In 2015, 24 children (“juniors,” 14 girls and 10 boys, aged M = 7.96 ± 0.69) and 22 parents (“seniors,” 14 mothers aged M = 38.86 ± 2.96 and 8 fathers aged M = 37.38 ± 2.97) were voluntarily enrolled in a study spread across three primary schools in the city of Poznań, Poland. The effectiveness of the intervention was determined according to postintervention behavioural changes in PA in comparison to preintervention levels, as reported by the parents and children. Overall, the study found increases in PA levels and reductions in sedentary time. Although the changes are modest, there are some unrecognized benefits of the intervention which may have occurred, such as improved sport and motor skills, more frequent family social behaviours (walks, meals, and visiting relatives), or simply improved quality of “do-together” leisure time PA.

## 1. Introduction

Regular PA gives a number of positive health benefits and reduces the risk of many diseases in children and youth [1–3] and as well in adults [4]. At the same time, the negative health consequences of being physically inactive have been identified in a number of studies [5–7]. PA guidelines vary, stipulating at least 60 minutes of moderate-to-vigorous physical activity (MVPA) per day for children and at least 150 minutes per week for adults [8].

Health behaviour habits in childhood tend to continue into adulthood. Likewise, decreased PA, insufficient physical fitness, and obesity also persist when moving from childhood into adulthood [9]. The school setting as a basis for PA interventions seems advantageous due to the fact that children from all risk groups and all segments of the population go to school. As such, schools have been singled out as being potentially effective sites for preventive strategies and promotion of a healthy lifestyle [10]. Interventions aimed at changing PA or sedentary behaviours are usually purposive courses aimed at promotion of positive changes in one or multiple factors influencing one's lifestyle (e.g., motor skills, psychological health, beliefs, knowledge, social context, and/or environmental opportunities for practicing the new behaviour) [11]. However, it is unclear for the educational system whether or not interventions, or indeed physical education (PE) in general, should focus on long-term aims increasing health awareness gradually. Both could eventually lead to a self-defined need to maintain physical fitness at a healthy level throughout life. As mentioned above, increased physical fitness and changes of actual health behaviours in children are expected to continue into adulthood, but this does not imply that such changes are not possible in adulthood.

In research on school PE, Møller et al. [10] found that an increase in the number of PE lessons from 2 to 6 hours a week does not change the total level of PA in primary school children when compared to controls (2 hours a week). Interestingly, children who had more PE classes were less active and less involved in organized leisure time sport participation during weekdays than their peers who had fewer PE contact hours. The authors [10] concluded that the difference may be caused by parents perceiving the children from extended PE groups as being sufficiently active in school and therefore skipping the efforts of facilitating transportation or other organizational aspects during leisure time.

Lai et al. [12] found that a sustainable outcome from interventions in children and adolescents is likely to occur in cases when an intervention is based on a theoretical model and lasts longer than 1 year. Recent studies have been focused on a wider array of ecological factors. McLeroy et al. [13] and Richard et al. [14] argue that the socioecological model helps to identify opportunities to promote PA by recognizing individual (e.g., attitudes, beliefs, and sex), behavioural (active/sedentary lifestyle), social environmental (parents and peers support), and physical environmental (availability of PA equipment and facilities) factors that may influence one's ability to be sufficiently physically active (skills and self-efficacy) and at the same time define potential determinants of health behaviours (including PA behaviours) [15]. PA participation (and generally health-related behaviours) is believed to be improved when environments support individual choices leading to better health. Therefore, to help us identify factors related to PA participation in a specific population (parents and children) in the supportive environment of the school setting, the present study was based on the social-ecological model [16]. The model describes influences on behaviour as a set of four interconnecting layers (individual, social, physical, and policy), all of which are embedded within each other. Since it is the individual who is at the centre of the model, in this paper we have concentrated on changes of selected personal factors (motor skills and self-esteem) and their associations with MVPA. Strategies which bring change at the individual level tend to focus on and include education and mentoring programs; therefore we used a school-based program.

Movement competency is assumed to be able to start/restart PA participation in children, adolescents, and adults [17]. Holfelder and Schott [18] found strong evidence from cross-sectional studies of a positive relationship between fundamental motor skills and organized PA. Research by Barnett et al. [19] reveals the cause-effect relationship between, on one hand, perceived sport competences and motor skills and, on the other hand, proficiency with the level of PA and fitness in children and adolescents. In a 5-month intervention program with 6-7-year-old children, Jarani et al. [20] found that exercise-based PE is more effective in improving gross motor function and cardiorespiratory fitness than games-based activity, which may be explained by a more regular and repetitious exercise-based mode than in the playing one. This may give some short-term effects and an increase in physical fitness but may be insufficient in bringing about changes in long-term personal beliefs and attitudes towards PA.

The most successful public health programs have been based on an understanding of health behaviours and the contexts in which they occur [21]. The present study aimed to evaluate changes in fitness parameters and selected PA aspects of both children and their parents. It was hypothesized that extended parental influence during playing time in form of “do together” PA with children would cause positive changes in MVPA and other fitness-related parameters. The intervention was part of the larger DEDIPAC project (Determinants of Diet and Physical Activity Knowledge Hub, number 4/JPI HDHL DEDIPAC KH/2014), which was the first research project of the European Union's Joint Programming Initiative on Healthy Diet and Healthy Life (JPI-HDHL).

## 2. Methods

### 2.1. Participants

In 2015, 24 children (“juniors,” 14 girls and 10 boys aged M = 7.96 ± 0.69) and 22 parents (“seniors,” 14 mothers aged M = 38.86 ± 2.96 and 8 fathers aged M = 37.38 ± 2.97) were voluntarily enrolled in a study in three primary schools in the city of Poznań, Poland.

Written consent was obtained from all parents (or carers) of all children participating in the program. Parents were also informed about the anonymous and voluntary nature of their participation, the fact that the study records would be kept confidential, and the fact that their individual contributions would be unidentifiable in the final report. Body mass and height data were collected by trained personnel with the use of anthropological instruments. Body height was measured to the nearest 0.5 cm using a portable stadiometer, and body mass was measured to the nearest 0.1 kg using electronic scales (Tanita Corporation, Japan). Detailed data are presented in Table 1. The study protocol was approved by the Local Bioethics Committee of The Karol Marcinkowski University of Medical Sciences in Poznan (decision number 947/14). Pretest and posttest examinations were undertaken prior to and immediately after the end of the program. All questionnaires (screening tool) were administered separately to parents and children, with the latter being helped on a “one-to-one” basis by a trained member of the staff and in comfortable conditions for a child (away from their parent).

### 2.2. Motor Fitness

Fitness was measured with the use of a 20-meter shuttle run test from the Eurofit battery of tests [22], also known as the Bleep or Beep Test. The endurance test was used to measure maximal running aerobic fitness. This test involved continuous running between two lines 20 meters apart in time to recorded beeps. The test subjects began running on the signal instructed by a remote source (tape). The subjects continued running between the two lines, turning when signaled by the recorded beeps. The test was stopped if the subject failed to reach the line (within 2 meters) for two consecutive ends. The result was the level (minute) in which the participant stopped running.

A sit and reach test measured the flexibility of the lower back and hamstring muscles. The test involved sitting on the floor with legs out straight in front. Feet were placed with the soles flat against a box, shoulder-width apart. The subject's task was to reach forward along the measuring line as far as possible. After one practice reach, the second reach was after at least two-second rest and the better result was recorded.

The purpose of the handgrip strength test was to measure grip or forearm muscle strength. The subject held the dynamometer in the dominant hand, with the arm at right angles and the elbow by the side of the body. The subject squeezed the dynamometer with maximum isometric effort, which was maintained for approximately 5 seconds. The subject had two trials and better result was recorded. The result was the maximum strength in Kg.

The purpose of the sit-up test was to measure the endurance of the abdominal and hip flexor muscles. The task of this test was to perform as many sit-ups as possible in 30 seconds. The test was performed on a mat with knees bent at right angles and with feet flat on the floor and hooked underneath a gym ladder. The fingers were interlocked behind the head. The result was the maximum number of correctly performed sit-ups in 30 seconds.

### 2.3. Physical Activity

The level of MVPA was determined with a Physical Activity Screening Measure [23]. One of the reasons for using this measure was its ease of application in school settings. Reliability was established at ICC = 0.77 and validity *r* = 0.40. This measure was comparable to those reported in other literatures [24] and used earlier in a population study in Poland [25]. The MVPA measure has also been used in a Health Behaviour in School-Aged Children study [24]. This measure corresponds to the average number of days per week with at least 60 minutes spent undertaking various forms of PA during which, in the participants' subjective opinion, their heart rates increased and they experienced a feeling of shortness of breath (higher breathing frequency). Participants were asked to answer two questions: *P*1: over the past 7 days, on how many days were you physically active for a total of at least 60 minutes per day?; *P*2: over a typical or usual week, on how many days are you physically active for a total of at least 60 minutes per day?

The MVPA index was calculated based on the following formula: MVPA = (*P*1 + *P*2)/2, where MVPA = PA index; *P*1 is the number of physically active days during the past 7 days; *P*2 is the number of physically active days during typical (usual) week.

### 2.4. Lifestyle

In order to measure factors associated with lifestyle, selected questions from the Health Behaviour in School-Aged Children (HBSC) questionnaire were used [24]. Among the items used were questions concerning leisure time activities: how much time a day do you spend playing computer games, including using tablets and smartphones? The answers ranged from “not at all” to “7 hours a day” and were separated into school and weekend days. The questions were also concerning self-evaluation of one's own physical fitness: how do you estimate your fitness? The answers were (1) very good, (2) good, (3) average, and (4) below average. There was also a question about family relationships and about the frequency of activities undertaken by the family together: how often do you spend time with your family (1) watching films, (2) playing board/computer games, (3) eating meals, (4) going for a walk, (5) visiting places, (6) visiting relatives, (7) doing sports, and (8) seating and talking? Possible answers ranged from every day to most of the days of the week, once a week, less than once a week, and never.

### 2.5. Intervention

A 15-week “Junior for Seniors” program was designed to target and improve the perceived sports competence of both children and their parents and to reinitiate or increase the PA of a family unit. The focus was on the development of fundamental bodily skills and sport-specific skills adjusted to the age group. The idea was that the environment should be fun and challenging and that skills should be practiced through child-oriented playing, exercising, and small games. Intervention was divided into 5 sport activity types of 3 weeks each (Movement Plays and Games, Traditional Sports, Tennis Activities, Nordic Walking, and Fitness and Dance activities). There were two (45 minutes) sessions every week led by a trained instructor. Sessions took place in the afternoons in the sport facilities of the local school. Participation was free of charge and voluntary and no record of presence was kept. The only criterion was that both the child and the parent/parents had to participate together. Additionally, there was also one hour a week dedicated to various aspects of nutrition.

### 2.6. Statistics

To compare the results between two quantitative measures (pretest and posttest for motor variables and MVPA) in the whole group of children (boys and girls analyzed as a group), a dependent samples* t*-test was used. In the group of parents, where mothers were treated separately from fathers, a two-way ANOVA was employed to compare the quantitative measures (a between-group factor, sex, and a within-group factor, posttest-pretest). In order to compare the results of ordinal measures (“playing together” and “self-esteem of physical fitness”) a Wilcoxon test was used.

## 3. Results

Table 2 contains the descriptive statistics of the motor fitness items for children and parents (mothers and fathers separately).

The intervention significantly increased scores for “sit-ups” (*t* = −2.36, df = 23, and *p* < 0.05) and the “20-meter shuttle run” (*t* = −3.97, df = 23, and *p* < 0.001) and showed a significant decrease in the results of the “handgrip” (*t* = 15.76, df = 23, and *p* < 0.001) in the children's group.

For “sit and reach,” the intervention significantly increased scores in the whole group of parents (main effect: *F*(1.20) = 20.07; *p* < 0.001; eta-square = 0.50). The effect of interaction “sex x intervention” did not reach statistical significance (*F*(1.20) = 0.04; *p* > 0.05). This indicates relatively similar changes of values in both the mothers' and fathers' groups. The main effect of “sex” was not reported as significant (*F*(1.20) = 0.73; *p* > 0.05).

The intervention significantly increased “sit-up” scores in the whole group of parents (main effect: *F*(1.20) = 36.15; *p* < 0.001; eta-square = 0.64). The effect of interaction “sex x intervention” did not reach statistical significance (*F*(1.20) = 0.74; *p* > 0.05). This indicates relatively similar changes of values in the mothers' and fathers' groups. The main effect of “sex” was reported to be significant (*F*(1.20) = 10.23; *p* < 0.01; eta-square = 0.34) and generally higher values were observed in the fathers' group.

For the “20-meter shuttle run” the main effect of intervention and interaction (sex x intervention) was not reported to be significant (resp., *F*(1.20) = 3.08 and *p* > 0.05; *F*(1.20) = 0.30 and *p* > 0.05). The effect of sex did reach statistical significance (*F*(1.20) = 29.43 and *p* < 0.0001; eta-square = 0.59) and higher values were observed in the fathers' group generally.

The intervention significantly decreased “handgrip” scores in the whole group of parents (main effect: *F*(1.20) = 56.99; *p* < 0.001; eta-square = 0.74). The effect of interaction (sex x intervention) did not reach statistical significance (*F*(1.20) = 0.64; *p* > 0.05), which indicates relatively similar changes of values in the mothers' and fathers' groups. The main effect of “sex” was reported to be significant (*F*(1.20) = 51.99; eta-square = 0.72) and generally higher values were observed in the fathers' group.

The descriptive characteristics of the mean and standard deviation for MVPA and median for ordinal variables like playing together and self-esteem of physical fitness by children and parents in pretest and posttest are shown in Table 3.

In the children's group, the intervention significantly increased scores for MVPA (*t* = −2.11, df = 23, and *p* < 0.05) and playing together (marginally significant effect: *Z* = 1.93; *p* = 0.053) and significantly decreased scores for self-esteem of physical fitness (*Z* = 2.21; *p* < 0.05).

For parents, none of the effects of MVPA were significant (resp., intervention: *F*(1.20) = 0.01 and *p* < 0.05; sex: *F*(1.20) = 0.82 and *p* < 0.05; interaction: *F* = 0.03 and *p* > 0.05). Differences for playing together were marginally significant in the mothers' group (*Z* = 1.96; *p* = 0.0505) and not significant in the fathers' group (*Z* = 1.69; *p* > 0.05). However, the intervention significantly increased scores in the whole group of parents (*Z* = 2.54; *p* < 0.05). Differences were not statistically significant for self-esteem of physical fitness (mothers: *Z* = 1.34 and *p* > 0.05; fathers: *Z* = 0.00 and *p* > 0.05; in the whole group: *Z* = 0.91 and *p* > 0.05).

Spearman's correlation coefficients between playing together, self-esteem and motor fitness items, and MVPA in the children's group (pretest) are presented in Table 4. The correlation coefficient between playing together and MVPA was significant (*r* = 0.43; *p* < 0.05).

Table 5 shows Spearman's correlation between variables: playing together, self-esteem and motor fitness items, and MVPA in the children's group (posttest). Two correlation coefficients were found to be significant: between self-esteem of physical fitness and the 20-meter shuttle run (*r* = −0.56; *p* < 0.01) and between playing together and the sit-ups (*r* = 0.41; *p* < 0.05).

Table 6 shows the correlation coefficients in the parents' group. The correlation coefficients between self-esteem of physical fitness and the 20-meter shuttle run (*r* = −0.56; *p* < 0.05), between self-esteem of physical fitness and MVPA (*r* = −0.52; *p* < 0.05), between self-esteem of physical fitness and handgrip (*r* = −0.41; *p* = 0.0568), and between playing together and MVPA (*r* = 0.47; *p* < 0.05) were reported to be significant.

The correlation coefficients between variables, playing together, self-esteem and motor fitness items, and MVPA in the children's group (posttest), are presented in Table 7. The correlation coefficients between self-esteem of physical fitness and sit-ups (*r* = −0.47; *p* < 0.05) and between self-esteem of physical fitness and MVPA (*r* = −0.41; *p* = 0.0559) were reported to be significant.

## 4. Discussion

The aim of this study was to analyze changes in the selected functional characteristics in a group of children and their parents participating in an intervention within the DEDIPAC program. The level of fitness was measured with the use of four selected items from the Eurofit battery of tests. The assessment of the parameters of fitness was made in accordance with the generally accepted concept of health-related fitness [26].

Analyzing the results obtained by the children participating in the study, it should be noted that only in 2 variables in the posttest did children obtain significantly higher results: “sit-ups” and the “20-meter shuttle run.” For “handgrip,” the results were significantly lower, which could be explained by the fact that the idea of the intervention was fun and challenging (especially strong fun factor). Furthermore, the conducted intervention, which included 5 sport activity types, considerably influenced the overall fitness levels of participants without recourse to any special muscle-strengthening activities. The intervention also considerably influenced the improvement of trunk muscle strengths and the overall fitness levels of participating children. This fact may be related to the selection of forms of PA carried out during the intervention program. Nordic Walking, Movement Plays and Games, Traditional Sports, Tennis Activities, and Fitness and Dance are all examples of aerobic physical activities, involving the whole body and large muscle groups, and focused mainly on endurance and strength.

In the parents' group, posttest brought significantly better results for “sit and reach” and, just as in the children's group, for “sit-ups.” For the “20-meter shuttle run,” no statistically significant difference was observed in the results obtained by either the mothers' group or the fathers' groups in terms of both parts of the test. However, a slight increase in this parameter was observed in both mothers and fathers. In the measurement of static force (handgrip), the posttest results obtained were considerably lower for both women and men. A significant improvement in flexibility test results in the parents' group could be due to the fact that the planned, well thought out, and, most importantly, regular exercise regimen, undertaken within the framework of the intervention program, helped increase the useful range of motion in the examined joints. Due to the fact that flexibility is the hallmark of a particular joint or a group of joints, the variety of forms of physical exercise offered during the intervention program could certainly have led to an increase in the amplitude of the movement.

The PA level of children and their parents was determined by calculating the value of the MVPA index (the MVPA criterion = 7 days averaged). In the children group, 3.1 ± 1.3 (pretest) and 4.1 ± 1.9 (posttest) increase was significant. In the group of parents, this value amounted to 2.7 ± 2.0–2.7 ± 1.6 (mothers) and 3.3 ± 2.4–3.4 ± 1.7 (fathers). The majority of children and adolescents examined in Poland and throughout the world [26] do not reach the required level of PA (5 days/week), which tallies with the results of other researchers. A study conducted in the United States by Troiano et al. [27] confirms that approximately 58% of children aged 6 to 11 years and over 90% of adolescents aged 12 to 19 fail to meet the recommended 60 minutes of PA per day, based on estimates derived from objectively measured PA (i.e., accelerometer). Many studies have also highlighted that interest in PA decreases with age and affects both sexes [28, 29]. These observations are reflected in the attitude of children and their parents towards PA presented in the study. An increase in MVPA levels (significant for children) have brought unrecognized benefits of the intervention such as improved sport and motor skills and more frequent family social behaviours (walks, meals, and visiting relatives together). An improved quality of leisure time PA could also have occurred.

In accordance with the expectations of researchers, the subjective assessment of the participants' own physical fitness level among both children and their parents (in the pretest) correlated with participants' PA. Involvement in MVPA among participants who assessed their physical fitness as high was significantly higher compared to that among those declaring low physical fitness. This result should not be considered particularly positive and it confirms the thesis that still more than half of the children and young people with very good predisposition to exercise do not meet the recommended level of PA. A limitation of this analysis is the fact that the study only includes fitness as one selected determinant of PA. The variability of the described parameters is determined by many factors and this is why other determinants, not described in this work, could also add to the final outcomes.

According to Tate et al. [30], parents play an important role in shaping children's health behaviours and may do so through direct modeling (i.e., engaging in PA behaviours observed by children, “do together”), which increases the likelihood that children will emulate their parents' actions. A similar standpoint is presented by Davison et al. [31], Griffith et al. [32], and Trost et al. [33, 34]. Parents' levels of activity are generally believed to be among the strongest determinants of their child's activity patterns. Several studies of school-aged children have found positive correlations of PA within families [30, 32, 35, 36]. All the studies were based on self-reporting or parental reporting of PA.

Higher self-evaluation of the fitness level has also not been found in the children's group after the intervention. According to Zawadzka et al. [37], perceiving the improvement of fitness as very important is the main factor that increases children's and teenagers' chances of meeting recommended levels of PA. This chance increases threefold in comparison with a group of people who considered the improvement of fitness less important or unimportant. Likewise, youths who evaluate their performance as either good or very good are 2.2 and 3.8 times, respectively, more likely to be active, compared to those having achieved the average or lower than average level of fitness.

This research has shown that “do together” (children with parents) activities significantly increase the level of MVPA in children. This may indicate strengthening of family ties through jointly undertaken PA in their leisure time. However, this relationship has only been observed in pretest. Thus, the intervention in the test group did not have a significant impact on improving the parent-child relationship in terms of joint and active organizing of leisure time PA. This may be explained by the fact that the study had some limitations. First, the study sample (children and parents) was not representative. Sample sizes were relatively small, especially when grouped by distinct age group (children and parents). Therefore, generalizability to other populations may not be appropriate. Furthermore, the temporal order of the effects between parenting and child MVPA is unknown. It is possible that children who are more active engage their parents in PA in ways that boost parental encouragement. Although both parent and child fitness levels were measured objectively, both parents' and children's behaviours were assessed through self-reporting. There is a need to adopt more direct measures of PA in the future in such interventions (e.g., pedometers and accelerometers). Future research should also include the complete socioecological model, especially with the area of the policy (financial resources of the parents and sociodemographic factors).

## 5. Conclusions

PA is important for both children's and parents' health and lowers the risk of obesity, heart disease, and diabetes. With regard to significant changes in selected parameters of physical and psychosocial consequences of joint participation in leisure time activities of children and parents, the effects of the conducted intervention turned out to be inconsiderable. The research issues presented in this paper prove the necessity of conducting an analysis of this type through larger scale studies. The evaluation of the PA intervention in a larger number of schools, spread throughout more regions, is required before the results can be generalized. Furthermore, understanding these relationships would help strategies to be designed which motivate children and their parents to be active throughout their lives. However, based on the conducted study, it may be concluded that one important goal has been achieved: that of more frequent family social behaviours (walks, meals, and visiting relatives together) and improved quality of leisure time PA.

## Figures and Tables

**Table 1:** Descriptive statistics of program participants (mean and standard deviation).

	*N*	Body height [cm]		Body mass [kg]		BMI	
		Pretest	Posttest	Pretest	Posttest	Pretest	Posttest
Children	24	127.1 ± 6.0	128.5 ± 6.2	25.2 ± 4.8	26.5 ± 4.8	15.5 ± 2.4	16.0 ± 2.2
Mothers	14	165.5 ± 4.3	165.5 ± 4.5	67.6 ± 12.5	67.5 ± 12.9	25.2 ± 3.8	25.1 ± 3.9
Fathers	8	175.6 ± 7.2	177.5 ± 8.2	82.9 ± 13.5	83.4 ± 11.5	26.6 ± 3.9	26.2 ± 3.6

**Table 2:** Descriptive statistics (mean and standard deviation) for motor fitness items in pretest and posttest.

	*N*	Sit and reach [number]		Sit-ups [number]		20-meter shuttle run [min]		Handgrip [kg]	
		Pretest	Posttest	Pretest	Posttest	Pretest	Posttest	Pretest	Posttest
Children	24	17.5 ± 4.2	18.3 ± 3.8	16.2 ± 3.1	17.1 ± 2.6^*∗*^	3.0 ± 1.0	4.0 ± 1.5^*∗∗*^	11.8 ± 2.9	6.1 ± 2.1^*∗∗*^
Mothers	14	24.5 ± 5.5	27.6 ± 4.6^**∗***∗*^	15.4 ± 2.8	19.4 ± 2.5^*∗∗*^	2.6 ± 0.7	3.2 ± 0.6	35.6 ± 4.8	28.7 ± 5.2^*∗∗*^
Fathers	8	22.5 ± 5.5	25.9 ± 4.9^**∗***∗*^	19.0 ± 2.9	22.0 ± 1.5^*∗∗*^	5.7 ± 1.8	6.0 ± 2.4	56.9 ± 9.1	48.4 ± 9.3^*∗∗*^

Note: significant effects are indicated in bold: ^*∗*^ *p* < 0.05  and  ^*∗∗*^ *p* < 0.001.

**Table 3:** Descriptive statistics (mean and standard deviation for MVPA and median for ordinal variables, playing together and self-esteem) in pretest and posttest.

	*N*	MVPA [days/week]		Playing together [points]		Self-esteem of physical fitness [points]	
		Pretest	Posttest	Pretest	Posttest	Pretest	Posttest
Children	24	3.1 ± 1.3	4.1 ± 1.9^*∗*^	2.0	3.0	2.0	1.5^*∗*^
Mothers	14	2.7 ± 2.0	2.7 ± 1.6	2.5	3.0	3.0	3.0
Fathers	8	3.3 ± 2.4	3.4 ± 1.7	2.5	3.0	2.0	2.0

Note: significant effects are indicated in bold: ^*∗*^ *p* < 0.05. Higher self-esteem corresponds to lower values.

**Table 4:** Correlation coefficients between variables: playing together, self-esteem and motor fitness items, and MVPA in children's group (pretest).

Variables	*N*	*R*, Spearman	*p*
Self-esteem & sit and reach	24	0.10	0.6427
Self-esteem & sit-ups	24	0.17	0.4391
Self-esteem & 20-meter shuttle run	24	0.10	0.6529
Self-esteem & handgrip	24	0.06	0.7909
Self-esteem & MVPA	24	−0.29	0.1747
Playing together & sit and reach	24	−0.17	0.4315
Playing together & sit-ups	24	0.24	0.2594
Playing together & 20-meter shuttle run	24	0.28	0.1912
Playing together & handgrip	24	−0.14	0.5115
Playing together & MVPA	24	0.43	**0.0380**

**Table 5:** Correlation coefficients between variables: playing together, self-esteem and motor fitness items, and MVPA in children's group (posttest).

Variables	*N*	*R*, Spearman	*p*
Self-esteem & sit and reach	24	0.06	0.7841
Self-esteem & sit-ups	24	−0.18	0.3963
Self-esteem & 20-meter shuttle run	24	−0.56	**0.0042**
Self-esteem & handgrip	24	−0.17	0.4334
Self-esteem & MVPA	24	−0.32	0.1263
Playing together & sit and reach	24	0.24	0.2599
Playing together & sit-ups	24	0.41	**0.0487**
Playing together & 20-meter shuttle run	24	0.10	0.6551
Playing together & handgrip	24	0.32	0.1247
Playing together & MVPA	24	0.19	0.3635

**Table 6:** Correlation coefficients between variables: playing together, self-esteem and motor fitness items, and MVPA in parents' group (pretest).

Variables	*N*	*R*, Spearman	*p*
Self-esteem & sit and reach	22	−0.18	0.4334
Self-esteem & sit-ups	22	−0.30	0.1739
Self-esteem & 20-meter shuttle run	22	−0.52	**0.0139**
Self-esteem & handgrip	22	−0.41	0.0568
Self-esteem & MVPA	22	−0.52	**0.0127**
Playing together & sit and reach	22	0.20	0.3637
Playing together & sit-ups	22	0.04	0.8528
Playing together & 20-meter shuttle run	22	0.20	0.3679
Playing together & handgrip	22	−0.08	0.7237
Playing together & MVPA	22	0.47	**0.0275**

**Table 7:** Correlation coefficients between variables: playing together, self-esteem and motor fitness items, and MVPA in parents' group (posttest).

Variables	*N*	*R*, Spearman	*p*
Self-esteem & sit and reach	22	−0.22	0.3293
Self-esteem & sit-ups	22	−0.47	**0.0278**
Self-esteem & 20-meter shuttle run	22	−0.41	0.0609
Self-esteem & handgrip	22	−0.34	0.1267
Self-esteem & MVPA	22	−0.41	0.0559
Playing together & sit and reach	22	0.28	0.2042
Playing together & sit-ups	22	−0.09	0.6816
Playing together & 20-meter shuttle run	22	0.19	0.3956
Playing together & handgrip	22	0.12	0.6022
Playing together & MVPA	22	0.30	0.1678
